# Autophagy in disease: hunger for translation

**DOI:** 10.1038/s41419-019-1419-2

**Published:** 2019-02-17

**Authors:** Francesca Pentimalli

**Affiliations:** Oncology Research Center of Mercogliano (CROM); Istituto Nazionale Tumori, IRCCS, Fondazione G. Pascale, Napoli, Italia

The last two decades have witnessed a tremendous progress in the autophagy field, spanning from the dissection of the molecular mechanisms governing selective autophagy to the subsequent identification of new strategies to modulate this process, along with the development of practical tools to assess autophagy and measure with extreme precision the autophagic flux. Overall, autophagy has increasingly emerged as a highly complex, context-dependent, and finely tuned process, which integrates with the other cellular pathways to maintain homeostasis in spite of physiological changes or stress cues and its dysfunction correlates with many pathologic conditions. So, while in the near horizon we can reasonably expect a blast of new possible applications of autophagy modulation for the treatment of ageing and an ever increasing number of diseases, the road to real breakthroughs still presents many hurdles.

In a recent focus issue, *Cell Death & Differentiation* collects a set of commissioned reviews by leading experts in the field to discuss new advances, the challenges ahead, and potential novel opportunities for targeting autophagy and expand its range of applications.

Of paramount importance for CDD readers is the link between autophagy and cell death. Autophagy is often induced by the same stress cues that lead to cell death too. But, is this just an accompanying phenomenon resulting from a cell effort to face harsh conditions? Well, it seems so in some cases, however, autophagy can also trigger cell demise, act in concert with other regulated cell death pathways and function independently of apoptosis or necrosis directly orchestrating the end, a process defined as autophagy-dependent cell death^1^. It is hence intuitive that, if we are to implement the clinical practice with approaches targeting autophagy, it is crucial to discern whether the outcome of autophagy modulation will be cell survival or demise. Denton and Kumar provide a nice overview of the possible contributions of autophagy to cell death^2^. They discuss the physiological role of autophagy in getting rid of obsolete tissues, such as the mid-gut or the salivary glands during the *Drosophila* larval-pupal transition, and analyze the signals inducing autophagy-dependent cell death along with the function of the specific components of the autophagic molecular machinery in driving this process. Overall, the authors highlight how the regulation of autophagy-dependent cell death is highly contextual and therefore its determination needs to be rigorously assessed in specific tissues, following accepted standards^1^, and through the careful dissection of the genetic/molecular contribution of the various regulated cell death pathways. Further expanding on the controversial nature of lethal versus non-lethal autophagy, Kriel and Loos report on the dynamic nature of cell death, discussing the process from the perspective of the cell energetic state and ATP availability which varies during the response to stress depending on the type and length of the stimuli^3^. Autophagy initially contributes to the cell response to stress raising ATP levels, but these can in turn help to carry out the apoptotic programme if the stress is prolonged, whereas necrosis occurs upon excessive damage and ATP decline. A cross talk between autophagy and apoptosis takes place at multiple levels throughout this process with autophagy being able to modulate the intrinsic pathway also through selective mitophagy. The authors elaborate on the concept of an autophagic flux threshold that, once breached, elicits cell death. Likely, the high ATP levels due to a ‘lethal’ autophagic flux can induce autosis, a particular autophagy-dependent cell death type that relies on the plasma membrane Na^+^/K^+^-ATPase^1^, the activation of which could be envisioned as an attempt to compensate high ATP production. Determining the autophagic flux in specific tumors, and inducing autosis through this mechanism, might be a new promising strategy against cancer^3^.

Further focusing into the cancer setting, Cecconi and colleagues delve into the role of autophagy in cancer stem cells (CSCs), a subpopulation of cells characterized by an high capacity to self-renew, differentiate, metastasize, and resist to conventional anti-tumoral treatments^4^. Autophagy acts as an adaptive mechanism to allow CSC survival and maintenance within their peculiar niche with mitophagy, in particular, playing a part into their metabolic rewiring. Also, the authors highlight how autophagy affects CSC capacity to migrate and invade through the modulation of epithelial-to-mesenchymal transition and the potential of autophagy modulation for counteracting both CSC chemoresistance and ability to evade immune recognition^4^.

In addition to CSCs, also endothelial cells (ECs), the main components of the blood vasculature, rely on autophagy to adjust their bioenergetic and biosynthetic needs in response to environmental changes. Agostinis and colleagues here provide a timely and comprehensive review covering the multifaceted role of autophagy in EC biology including effects on vascular ageing, permeability and vasodilatation, hemostasis and lipid homeostasis, the latter occurring through selective lipophagy, which protects against atherosclerotic plaque formation^5^. The protective effect of autophagy in this context is highly relevant as epitomized by two main facts: first, long-term calorie restriction slows down vascular ageing; second, autophagy is compromised in the aged endothelial compartment concomitantly with an increased risk of cardiovascular disease. The authors also analyze EC-associated autophagy in pathological angiogenesis, particularly in tumorigenesis, discussing both cell autonomous roles (in regulating redox homeostasis and metabolic rewiring) and emerging non-cell autonomous functions realized through the secretion of factors acting as autocrine and paracrine factors^5^. Delineating these complex functions will be instrumental to the design of more effective strategies targeting angiogenesis in cancer.

The autophagy and ageing connection is well established also with regards to cardiac dysfunction. In this issue, Miyamoto discusses the main features of the ageing heart and the possible molecular mechanisms whereby the autophagy decline observed with advanced age affects the functions of this high-energy demanding organ. The author provides clues as to how autophagy modulation could be achieved to improve cardiac function thereby preventing cardiovascular diseases, which is the leading cause of death worldwide^6^.

Papandreou and Tavernarakis zoom into the selective process of nuclear recycling – nucleophagy—described under basal or nutrient-deprived conditions in yeast and associated to pathological states such as cancer and degeneration. The authors discuss the possible role of basal nucleophagy in humans and how its fine-tuning might be used to prevent senescence and age-related diseases^7^.

Besides its key metabolic functions and the quality control role on organelles and unnecessary or aggregated macromolecules, autophagy has emerged also as crucial to multiple aspects of the immune response, which extend beyond xenophagy. In two companion articles within this focused issue, Simon and coauthors survey numerous autophagy-related mechanisms that impact on the immune system activities. They show the relevance of autophagy in regulating innate immune cells such as neutrophils, eosinophils, mast cells, and NK cells influencing phagocytosis, differentiation, degranulation, cytokine release, and extracellular trap formation^8^. Whereas in another article they focus on monocytes/macrophages and dendritic cells discussing how autophagy impacts on phagocytosis, antigen presentation, cytokine production, control of inflammasome activation, and tolerance^9^, overall suggesting that autophagy modulation will offer new opportunities for antimicrobial defence, treatment of inflammatory, and other diseases in which the immune system plays a key part.

Finally, in another must-read article, Maiuri and Kroemer challenge the readers with a provocative question: the tremendous knowledge accumulated in recent years inched us closer to the application of therapeutic strategies based on autophagy modulation, but which disease will be the first to be treated in this wide scenario? They discuss how inhibiting autophagy could be beneficial in advanced cancers or to reduce cell death in some settings. Conversely, inducing autophagy could counteract ageing, obesity, cardiovascular disease, antitumoral immunosurveillance, and also neurodegeneration and other diseases. Likely, targeting selective autophagy rather than general mechanisms will have fewer side effects considering the Janus nature of autophagy and that core autophagy effectors have also other unrelated functions^10^. Rubinsztein and colleagues, for example, discuss how the different post-translation modifications of Beclin 1, a key factor of the autophagic pathway, could offer the opportunity to fine-tune autophagy more specifically in certain contexts^11^.

At last, as some well-established effects of natural compounds or ‘old’ drugs have been now explained through their action on autophagy, will this be enough to favor their repurposing or translation to the clinic even without biopharma interest and support? We still have not answered all the questions put forward by Maiuri and Kroemer^10^, but we bet that we will not starve long for effective autophagy-based translational approaches.
